# Cold-Curing Structural Epoxy Resins: Analysis of the Curing Reaction as a Function of Curing Time and Thickness

**DOI:** 10.3390/ma7096832

**Published:** 2014-09-22

**Authors:** Carola Esposito Corcione, Fabrizio Freuli, Mariaenrica Frigione

**Affiliations:** Department of Engineering for Innovation, University of Salento, 73100 Lecce, Italy; E-Mails: ffreuli@gmail.com (F.F.); mariaenrica.frigione@unisalento.it (M.F.)

**Keywords:** cold-cured epoxy resin, curing, frontal polymerization

## Abstract

The curing reaction of a commercial cold-curing structural epoxy resin, specifically formulated for civil engineering applications, was analyzed by thermal analysis as a function of the curing time and the sample thickness. Original and remarkable results regarding the effects of curing time on the glass transition temperature and on the residual heat of reaction of the cold-cured epoxy were obtained. The influence of the sample thickness on the curing reaction of the cold-cured resin was also deeply investigated. A highly exothermal reaction, based on a self-activated frontal polymerization reaction, was supposed and verified trough a suitable temperature signal acquisition system, specifically realized for this measurement. This is one of the first studies carried out on the curing behavior of these peculiar cold-cured epoxy resins as a function of curing time and thickness.

## 1. Introduction

Epoxy resins are commonly used in civil engineering applications and in cultural heritage conservation due to their advanced chemical and mechanical properties, as repairing materials, adhesives, coatings, and matrices for composites. For such applications, practical and economical reasons force the use of “cold-curing” resins, that is, epoxy systems able to achieve a suitable degree of cure and acceptable mechanical properties in reasonable curing times when cured at ambient temperatures [1,2,3,4,5,6,7]. Aliphatic amines are usually used as curing agents for this purpose, since they are able to react with epoxies also at low temperature. However, many weeks of cure, if compared to the few hours of curing time required by the epoxies, cured with a source of heat, are necessary to provide a material with a satisfactory degree of cross-linking. Nevertheless, the conversion of epoxy groups is never complete and a moderate glass transition temperature (Tg), which generally does not exceed 60 °C [6,8,9,10,11,12], is obtained. As a result, the exposure to a temperature slightly higher than ambient temperature is likely to cause the restart of cross-linking reactions, with a consequent increase in Tg and, in turn, the stiffening of the system. On the other hand, the absorption of external water, both in liquid or vapor form, produces a decrease in the initial Tg of the resin, which affects the mechanical properties and may enable a post-curing process, even at these low environmental temperatures [13].

These thermodynamically unstable systems can also undergo to physical aging: it leads to a reduction in the polymer’s free volume over time (namely a “densification” process), with a consequent modification of all of the mechanical, temperature-dependent properties [14,15,16]. Physical aging in the glassy state is very slow, whereas it proceeds rapidly at temperatures close to the Tg. This latter is the case of cold-cured resins, in which Tg can be easily approached and even exceeded by the external temperature [17]. Physical aging is a thermo-reversible phenomenon, which can be erased when the polymer is heated above its Tg. When the service temperature exceeds the Tg of aged cold-cured epoxy, the “rejuvenation” of the resin takes place, *i.e.*, the erasing of physical aging with the recovering of the initial properties. Cold-cured epoxy resins exposed to natural weather, therefore, are constantly subjected to aging and de-aging processes that take place in non-isothermal conditions, depending on the actual meteorological weather [18].

Starting from the peculiar behavior of cold-curing epoxies, previously described and discussed, it is clear that there is a pressing need to find reasonable tools to ascertain the curing reactions and the cross-linking degree of commercial cold-cured epoxy-based resins, representing the most frequently used polymeric materials for applications in civil engineering. The durability of these systems when exposed to environmental agents, in addition, is strongly connected to their curing reaction. The latter influences, not only the physical-mechanical properties of the resin, but also its performance when reinforced with proper nanofillers, potentially able to improve its properties. 

In a previous paper [19], the same authors analyzed the influence of graphene stacks nano-platelets on the glass transition temperature and flexural properties of a commercial cold-cured epoxy resin. They demonstrated that the presence of nanofillers was not able to improve the properties of the neat resin, as it would be expected. A possible explanation of this surprising behavior was hypothesized by supposing that the neat epoxy resin is able to polymerize by a frontal polymerization process and that this latter is slowed in presence of an inert material, such as the graphene stacks precursor, due to the high dissipated heat [20]. In the present study, this hypothesis would be confirmed investigating the curing reaction of the cold-curing epoxy as a function of the curing time and thickness.

## 2. Experimental Section

### 2.1. Materials 

The material object of this study is a commercial epoxy adhesive produced and supplied by MAPEI S.p.A. (Mettler Toledo, Milano, Italy). The system is representative of epoxy resins used as matrix for fiber reinforced composites (FRP) in rehabilitation procedures and it has been already widely employed for restoration of both civil and monumental buildings [21].

The commercial epoxy resin is a diglycidylether of bisphenol-A (indicated as component A), while the curing agent is a mixture of aliphatic and aromatic amines, *i.e.*, polyethylenimine-m-xylenediamine-nonylphenol (indicated as component B), provided ready-to-use. Nonylphenol is usually added in order to increase the rate of curing of the mixture due to the presence of phenolic OH-group. The nonyl group, as an aliphatic chain, can also reduce the evaporation during the application procedure and it has plasticizing effects as well.

Samples of the resin were prepared using the mixing ratio suggested by suppliers, that is resin:hardener = 4:1 by weight. The exact amount of each component was weighed with an analytical balance with an accuracy of ±0.1 mg. The hardener was poured into the base resin and they were gently stirred, avoiding the formation of air bubbles, until the mix was perfectly homogeneous. Specimens of standard rectangular form (dimensions: 90 × 10 × 5 mm^3^) were obtained by pouring the mix into Teflon molds of rectangular shape. The specimens’ shape and dimensions were chosen on the basis of American Society for Testing and Materials (ASTM) standard for flexural mechanical tests [22].

The samples were cured at a temperature of 23 ± 2 °C and a relative humidity (R.H.) of 50% ± 5%. They were removed from the molds after 26 h and maintained under the same controlled conditions of temperature and humidity (*i.e.*, T = 23 ± 2 °C and R.H. = 50% ± 5%) for further 6 days, being 7 days the curing time reported by suppliers [23]. However, as previously reported, since it is well known that the curing time required to the commercial cold-curing epoxies to achieve the final properties is much higher than indicated by suppliers, the investigation of curing process was performed beyond 7 curing days. 

### 2.2. Experimental Techniques

The curing reaction of the cold-cured epoxy resin was analyzed at different curing times (ranging from 7 to 62 days) and thickness (ranging from 0.8 to 40 mm) employing a differential scanning calorimeter (DSC 622, Mettler Toledo, Italy). Each sample was heated from room temperature up to 250 °C at 10 °C/min under nitrogen atmosphere, performing at least three tests on each set of samples averaging the results. The glass transition temperature was determined as the transition midpoint; the relaxation enthalpy and the residual heat of cross-linking reactions were evaluated from the endothermic and exothermic, respectively, peak areas delimited by the tangent lines to DSC curve. The occurrence of physical aging in a polymer isothermally aged can be measured in a dynamic calorimetric experiment. An endothermic peak in the glass-transition region, whose position and intensity depends on temperature and time of aging, is, in fact, observed for aged polymers. The area of this peak is related to the relaxation enthalpy (ΔH_rel_) and it increases with the aging time, becoming constant when the equilibrium is achieved.

## 3. Results and Discussion

**Figure 1:**
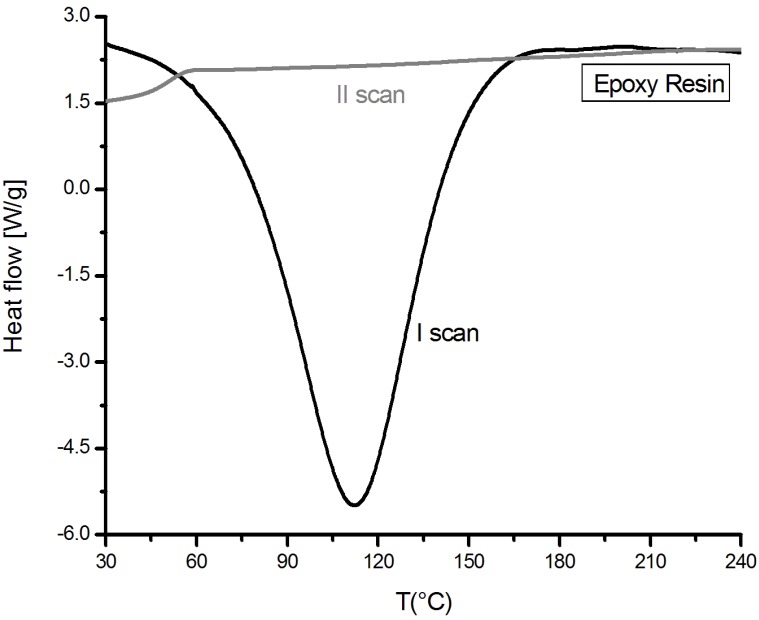
DSC thermograms (I and II scan) of the commercial cold-cured epoxy resin.

A DSC investigation was, first, performed on the epoxy-based liquid mixture obtained by mixing the components A (diglycidylether of bisphenol-A) and B (a mixture of aliphatic and aromatic amines, *i.e.*, polyethylenimine-m-xylenediamine-nonylphenol), as already described. In Figure 1, the thermograms obtained by two subsequent dynamic DSC scans performed on epoxy resin are reported.

From first DSC curve, it is evident that the cross-linking reaction is able to start at room temperature (around 30 °C) and it can be considered completed at about 200 °C. The reaction is highly exothermic, since it develops a heat of reaction of about 407 J/g. On the other hand, by analyzing in DSC the same samples in a second scan, no residual peak of reaction was found, evidencing that the reaction was completed after the first DSC scan. The fully cured epoxy system was able to achieve a maximum Tg of about 49 °C.

The curing reaction at ambient temperature was, then, analyzed by curing at room temperature (23 ± 2 °C) epoxy samples, with a thickness of 5 mm, for long times, ranging from 7 to 62 days. From the first and the second DSC scans, performed at different curing times, the glass transition temperature Tg (measured by using midpoint method), the residual heat of reaction ΔH_res_, the relaxation enthalpy (ΔH_rel_) and the Tgmax value, *i.e.*, the Tg of each sample fully cured in the first dynamic DSC scan, were measured. The results are reported in Table 1.

Referring first to the Tg values of the epoxy resin, reported in Table 1 as function of the curing time at ambient temperature, the epoxy resin achieves a Tg of about 47 °C after 7 days of cure. After 62 days of cure, the Tg increases of only 5 °C. This result is confirmed by the residual heat of reaction measured at the same curing time, which continuously decreases only 6 J/g from 7 to 62 days of cure. Furthermore, the relaxation enthalpy (ΔH_rel_), that represents a measure of the physical aging phenomenon, is almost constant with the curing time. We can conclude, therefore, that a curing time higher than 7 days has only a limited effect on the completion of the curing reaction, since there are moderate differences in terms of Tg and residual heat of reaction, even after 62 days. The DSC results are also in perfect accordance with the technical data sheet of the commercial resin, in which about 7 days is reported to be the advisable curing time [23].

**Table 1:** Tg_midpoint_, ΔH_Res_, ΔH_Rel_ and Tg_max_ values measured on cold-cured epoxy samples (thickness = 5 mm), cured at room temperature for different time spans, by dynamic DSC scans.

Curing time (days)	I scan			II scan
	Tg_midpoint_ (°C)	ΔH_Res_ (J/g)	ΔH_Rel_ (J/g)	Tg_max_ (°C)
7	46.9 ± 0.1	38.3 ± 6.2	6.0 ± 0.3	62.3 ± 3.7
14	48.0 ± 0.4	35.6 ± 2.6	5.5 ± 0.8	62.5 ± 0.6
30	47.5 ± 0.8	33.1 ± 1.7	7.1 ± 1.6	63.9 ± 2.0
62	52.0 ± 1.3	31.8 ± 0.5	6.4 ± 0.3	61.2 ± 1.3

The Tg of the epoxy resin, even after 62 days, is rather low. An average Tgmax of about 62 °C could be, in fact, achieved only when the cure is completed (last row of Table 2). However, this condition was not yet attained after 62 days of curing at ambient temperature. As already mentioned, a low glass transition temperature (not much higher than 50 °C) could be responsible of a loss of mechanical properties of the resin and a lack of adhesion with the fibers and substrate [24].

The slight increase in Tg is a direct result of the slow proceeding of the cross-linking reactions occurring in the thermosetting (*i.e.*, epoxy) system, and its determination can be used to measure the extent of these reactions. The Tg was proved to be an accurate and sensitive parameter for monitoring the cross-linking reactions mainly at high conversion levels [25] As the cure proceeds, the Tg of the system increases and, at some time, it will exceed the cure temperature (ambient temperature), *i.e.*, the vitrification process occurs. At this stage, the mobility of the reactive groups is significantly reduced and the curing reactions are diffusion controlled, *i.e.*, they appreciably slow and finally stop; consequently, the value of the Tg tends to level off [26]. 

On the other hand, by comparing the values of residual heat of reaction of Table 2 with the total heat of reaction of Table 1 it is evident that the residual heat of reaction can be considered quite small, even after 7 days and that the cure can be considered almost near to be complete. This is also confirmed by the Tg measured for the epoxy resin completely cured at high temperature that is comparable with that measured on the resin cold-cured for different time spans (in Table 1).

In order to have an evidence of this consideration, the maximum extent of reaction (α) achieved by the cold-cured epoxy resin at each curing time was calculated as follows:

(1) $$\alpha =\frac{H_{0}-H_{t}}{H_{0}}$$

where: H_0_ is the heat of reaction measured from the first DSC scan performed on the liquid (un-cured) epoxy resin (about 407 J/g) and Ht is the residual heat of reaction measured from the DSC dynamic scan performed on the cold-cured epoxy at different curing times and reported in Table 1.

**Figure 2:**
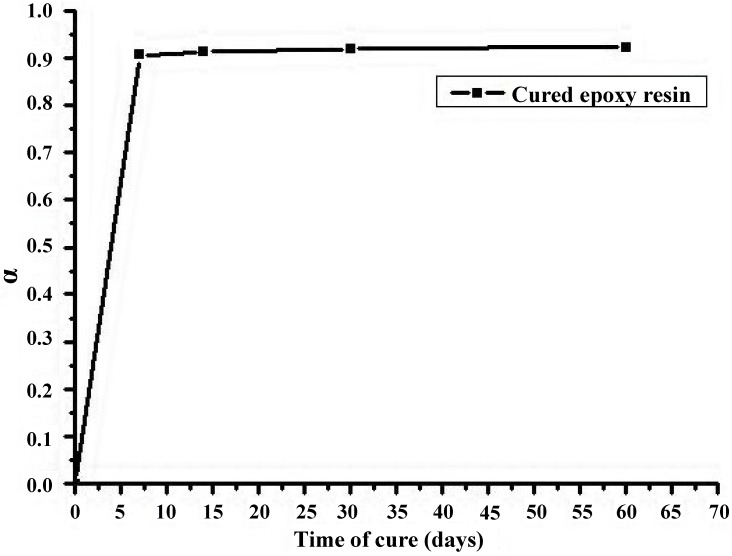
Extent of cross-linking reactions for the cold-cured epoxy resin as a function of the curing time.

The α values determined for the 5 mm thickness samples are reported as function of the curing time in Figure 2.

The data reported in Figure 2 evidence that the samples of 5 mm achieve an extent of reaction of about 90% after only 7 days of curing at ambient temperature (about 23 °C), and that this value remains almost unchanged up to 60 days.

**Figure 3:**
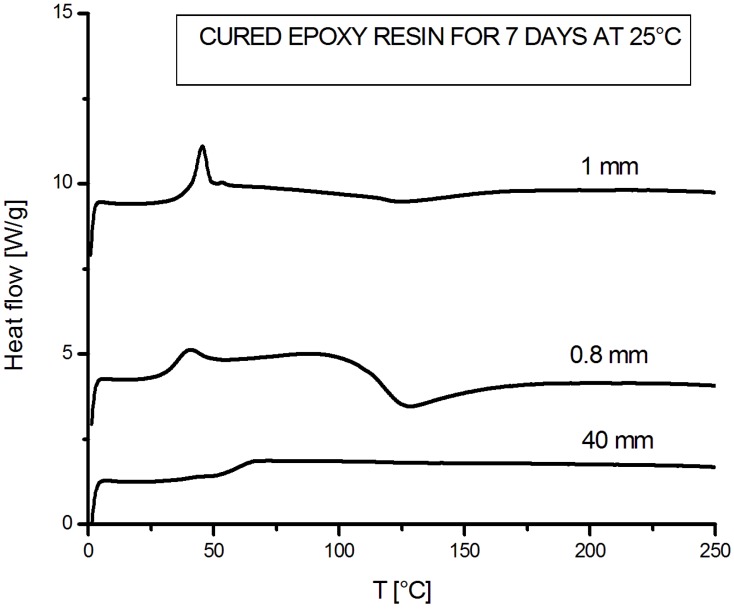
Thermograms obtained for the samples of 0.8, 1 and 40 mm thickness.

Further experimental analyses, performed by DSC dynamic tests, were finally devoted to understanding if the sample thickness could influence the curing reaction of the resin. To this aim, several cold-cured epoxy specimens, with thickness ranging from 0.8 to 40 mm, were produced, kept in laboratory at room temperature and characterized by dynamic DSC scans. In view of the fact that curing times longer than one week seem to have only a slight influence on the proceeding of the cross-linking reactions (see Figure 2), a curing time of 7 days was selected and used for the experimental measurement. The thermograms obtained for the samples possessing thickness of 0.8, 1 and 40 mm are reported in Figure 3.

From the observation of the DSC curves reported in Figure 3, it is clear that curves, relative to thin specimens (0.8 and 1mm), are very close and displays the Tg, the subsequent relaxation peak (relative to the de-aging process) and a residual heat of reaction. On the other hand, in curve, relative to the thicker specimen (40 mm), only a Tg was measurable, much higher than that measured on thinner specimens. 

The average values of glass transition temperature Tg (measured by using the midpoint method), residual heat of reaction, ΔH_res_, relaxation enthalpy, ΔH_rel_, and Tg_max_, *i.e.*, the Tg of each fully cured sample (measured in a subsequent dynamic DSC scan), are reported in Table 2 as function of the sample thickness. For each specimen, the external part of the sample was taken and analyzed in DSC.

**Table 2:** Tgmidpoint, ΔH_res_, ΔH_rel_ and Tg_max_ values measured by dynamic DSC scans for 7-day cold-cured samples, as function of the thickness of the specimen.

Thickness (mm)	Tg (°C)	ΔH_Rel_ (J/g)	ΔH_Res_ (J/g)	Tg_max_ (°C)
0.8	33.3 ± 0.7	7.1 ± 0.1	72.6 ± 1.1	63.1 ± 1.5
1	42.5 ± 0.3	7.0 ± 0.4	47.5 ± 1.9	65.5 ± 1.7
5	46.9 ± 0.9	6.0 ± 0.6	38.3 ± 0.9	65.3 ± 1.3
10	44.2 ± 1.1	3.3 ± 0.2	21.1 ± 1.3	66.6 ± 1.6
40	56.4 ± 0.7	–	0.9 ± 0.6	63.2 ± 1.5

The actual influence of the sample thickness on the curing reaction of the commercial cold-cured epoxy resin can be deduced by the experimental DSC data reported in Table 2. ΔH_Res_ and ΔH_Rel_ both decrease and Tg increases by increasing the sample thickness; furthermore, all samples possess roughly the same Tg_max_ when completely cured. In particular, the sample with a thickness of 40 mm does not exhibit physical aging phenomenon, at least up to one week of curing, showing a null value of the relaxation enthalpy. This is due to a Tg (about 56 °C) higher than the curing (ambient) temperature. In such conditions (Tg > T), the relaxation process does not proceed. Furthermore, the same sample shows a quasi-null residual heat of reaction (about 1 J/g), evidencing that in this case the curing reaction is almost complete. These results seem, therefore, to suggest that the reaction is favored by a high thickness.

A possible explanation of this experimental evidence can be attributed to the high exothermicity of the curing (cross-linking) reactions of the epoxy resin. By increasing the thickness of the sample, the developed heat of reaction increases, and this allows to obtain a greater degree of cross-linking and, consequently, a higher Tg of the sample. This kind of polymerization reaction could be identified as “frontal polymerization” and allows to convert a monomer into a polymer exploiting the exothermicity of the self-same polymerization reaction. If the heat dissipated is not excessive, the quantity left over may be sufficient to induce the polymerization of the monomer layer close to the zone heated by the reaction. As a result, a hot propagating and self-sustaining front can be observed in a very thick sample [27,28,29,30,31]. 

In order to have an experimental evidence of the possible occurrence of a frontal polymerization in the cold-cured epoxy resin, a temperature signal acquisition system, interfaced with a proper computer and two thermocouples (type K), was placed above the propagating front in a specimen possessing a very high thickness. To this aim, an amount of un-cured resin/hardener mix of about 154 cm^3^ of volume was poured into a cylindrical glass container, with a diameter of 7 cm. The two thermocouples were placed on the surface and in the bulk of the resin, respectively, and the curing reaction proceeded at ambient (laboratory) temperature. 

**Figure 4:**
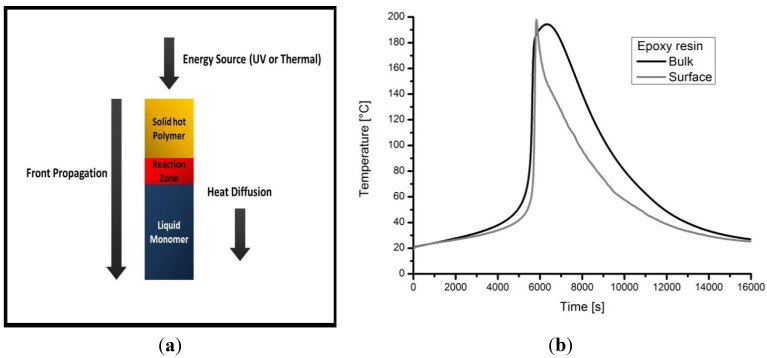
(**a**) Schematic representation of the frontal polymerization reaction; (**b**) temporal temperature profiles from the two thermocouples placed in the bulk and on the surface of the liquid resin.

A schematic representation of the possible frontal polymerization reaction and the temporal temperature profiles obtained from the two thermocouples placed in the bulk and on the surface of the epoxy 40 mm thickness sample are reported in Figure 4a,b respectively.

The temporal temperature profiles, reported in Figure 4b, confirm that the resin used in this study is able to polymerize with a reaction that is self-activated at room temperature and, then, it could be considered a frontal polymerization reaction. From the observation of the curves reported in Figure 4b, it can be deduced that there is a significant period (up to 6000 s) during which the temperatures registered by the two thermocouples are almost identical, indicating that the cross-linking reactions, during the initial heating process, proceed in the same way on the surface and in the bulk of the mix. At longer curing times, the two curves are substantially different, indicating a different behavior on surface an inside the 40 mm thickness specimen. The isothermal period of the sample includes the time of the front’s propagation, as well as a significant portion of the seed dissolution time (or induction period). Thus, the front can be considered isothermal, which indicates that the surrounding air and the monomer above the front are sufficient for dissipating the heat produced from the exothermic reaction of the front and the slower bulk polymerization of the monomer. 

## 4. Conclusions

A deep calorimetric analysis was performed in order to establish the influence of the curing time and the sample thickness on the cross-linking reaction of cold–curing epoxy resins, used as matrix for FRP composites and/or adhesive in civil engineering applications. The experimental data obtained on a commercial cold-cured epoxy, already employed as matrix for FRP strengthening systems, show that a time of about seven days is enough to almost complete the curing reaction at ambient temperature. A maximum extent of reaction of about 0.9 was, in fact, obtained in 5 mm thickness-samples after seven days of curing, remaining the extent of reaction almost unchanged up to 60 days. The influence of the sample thickness on the cure reaction of the resin was also investigated by DSC analysis, obtaining a clear evidence of the positive effect of the thickness on the curing degree of cross-linking reactions. A possible explanation of this experimental evidence was the high exothermicity of the curing reaction of the epoxy resin: it was supposed, in fact, that by increasing the thickness of the sample, the heat of reaction developed increases, obtaining greater degrees of cross-linking and, in turn, higher Tg’s. 
